# Achieving global mortality reduction targets and universal health coverage: The impact of COVID-19

**DOI:** 10.1371/journal.pmed.1003675

**Published:** 2021-06-24

**Authors:** Wenhui Mao, Osondu Ogbuoji, David Watkins, Ipchita Bharali, Eric Nsiah-Boateng, Mohamed Mustafa Diab, Duah Dwomoh, Dean T. Jamison, Preeti Kumar, Kaci Kennedy McDade, Justice Nonvignon, Yewande Ogundeji, Fan-Gang Zeng, Armand Zimmerman, Gavin Yamey

**Affiliations:** 1 Center for Policy Impact in Global Health, Duke Global Health Institute, Duke University, Durham, North Carolina, United States of America; 2 Department of Medicine, University of Washington, Seattle, Washington, United States of America; 3 National Health Insurance Authority, Accra, Ghana; 4 Department of Biostatistics, School of Public Health, College of Health Sciences, University of Ghana, Legon, Ghana; 5 Institute for Global Health Sciences, University of California San Francisco, San Francisco, California, United States of America; 6 Public Health Foundation of India, New Delhi, India; 7 Department of Health Policy, Planning and Management, School of Public Health, University of Ghana, Legon, Accra, Ghana; 8 Health Strategy and Delivery Foundation, Abuja, Nigeria; 9 Center for Hearing Research, University of California Irvine, Irvine, California, United States of America

## Abstract

Wenhui Mao and coauthors discuss possible implications of the COVID-19 pandemic for health aspirations in low- and middle-income countries.

**Summary points**

- The Coronavirus Disease 2019 (COVID-19) pandemic threatens progress toward a “grand convergence” in global health—universal reduction in deaths from infections and maternal and child health conditions to low levels—and toward achieving universal health coverage (UHC).
- Our analysis suggests that COVID-19 will exacerbate the difficulty of achieving grand convergence targets for tuberculosis (TB), maternal mortality, and, probably, for under-5 mortality. HIV targets are likely to be met.
- By 2035, our analysis suggests that the public sectors of low-income countries (LICs) would only be able to finance about a third of the costs of a package of 120 essential non-COVID-19 health interventions through domestic sources, unless the country increases significantly the priority assigned to the health sector; lower middle-income countries (LMICs) would likewise only be able to finance a little less than half.
- The likelihood of getting back on track for reaching grand convergence and UHC will depend on (i) how quickly COVID-19 vaccines can be deployed in LICs and LMICs; (ii) how much additional public sector health financing can be mobilized from external and domestic sources; and (iii) whether countries can rapidly strengthen and focus their health delivery systems.

## Introduction

In 2013, the Lancet Commission on Investing in Health (CIH) published Global Health 2035, a global health investment framework [1]. The CIH showed that with the right investments to scale up existing evidence-based health interventions and new health technologies, a “grand convergence” in global health—a universal reduction in deaths from infections and maternal and child health conditions to low levels—was possible by 2035. The CIH also showed the feasibility of sharply reducing the global burden of noncommunicable diseases (NCDs) and of reaching universal health coverage (UHC) with a package of “pro-poor” interventions.

This vision could now be under threat. The Coronavirus Disease 2019 (COVID-19) pandemic has led to disruption of prevention and treatment services worldwide [2,3], such as tuberculosis (TB) and childhood vaccination programs [4–6]. This article examines the potential impact of such disruptions on progress toward grand convergence and UHC. We begin with a brief summary of Global Health 2035 and of the follow-up report (“CIH 2.0”), published 5 years later in 2018, since these 2 reports provide a baseline upon which we can assess the impact of COVID-19. Next, we summarize evidence on how COVID-19 is affecting health services. We then analyze historical trends in maternal, child, HIV, and TB mortality from 2000 to 2019. We use these to explore what would have happened if these trends had continued unchanged (a “no COVID-19” scenario) and what might happen under 3 alternative COVID-19 disruption scenarios (minor, moderate, or severe disruption in long-term mortality trends). We examine how the pandemic’s impact on the global economy could affect the realization of UHC in low-income countries (LICs) and lower middle-income countries (LMICs). We conclude by exploring what our findings mean for domestic and international health policymaking.

### An opportunity to achieve universal mortality reductions and UHC by 2035

The CIH defined grand convergence as a universal reduction in deaths from infections and maternal and child conditions down to the levels seen in the best-performing middle-income countries [1]. This would mean achieving a maternal mortality ratio (MMR) of 64 per 100,000 live births, an under-5 mortality rate (U5MR) of 16 per 1,000 live births, an annual HIV death rate of 8 per 100,000 population, and an annual TB death rate of 4 per 100,000 population (“64–16–8–4”). Global Health 2035 showed that grand convergence could be achieved through aggressive scale-up of proven health interventions, strengthening health systems to deliver these interventions, and distribution of new health technologies [7].

Five years later, in 2018, the CIH published “Alma-Ata at 40 years: reflections from the Lancet Commission on Investing in Health” (CIH 2.0) [8], which provided a mixed picture on progress. The encouraging news was that if the global trends in mortality seen in 2010 to 2016 were to continue, the global convergence targets for under-5 and HIV mortality would be achieved around the year 2035. But the worrying news was that if the rates of decline for maternal and TB mortality remained similar to 2010 to 2016, the convergence targets would not be achieved until 2067 and 2074, respectively [9].

The CIH 2.0 report also found that most LMICs, except India, could afford an “essential UHC” package of 218 health interventions from domestic public financing. However, most LICs, and sub-Saharan Africa as a region, would not be able to afford even a “highest priority package” of 108 interventions delivered in primary care (S1 Text) without substantial increases in the priority they assign to health [10]. These countries would need external financing to make up for insufficient resources. Global Health 2035 was published at a time when the International Monetary Fund (IMF) was very optimistic about the pace of economic growth in LICs and LMICs. By 2018, the projections of such growth were less optimistic. Below, we consider the newest IMF projections and what these might mean for financing UHC in the pandemic and post-pandemic era.

Both CIH reports called for a reorientation of health aid toward “global functions”—activities with cross-national benefits, such as product development for neglected diseases and pandemic preparedness. In 2013, only 23% of all donor funding for health was directed toward global functions [11]. This proportion rose in the wake of West Africa’s Ebola outbreak, but, by 2017, it had fallen again [12], suggesting that donors are prone to cycles of “panic and neglect” in funding global functions.

### Threats to convergence and UHC: How COVID-19 is disrupting essential services

Above, we described a pathway to grand convergence and UHC based on aggressive scale-up of existing and new interventions through strengthened delivery systems. How might COVID-19 have affected such healthcare delivery in LICs and MICs? The illness has had a direct impact on health services: Clinics and hospitals in many hard-hit countries have been overwhelmed with patients with COVID-19. In addition, there are at least 4 main factors described in the literature that led to an indirect impact of COVID-19 on health services, although there are likely to be other more complex pathways that are yet to be delineated.

First, COVID-19 mitigation policies and patients’ fears of getting COVID-19 from healthcare settings affected service use. For example, during the first lockdown period in South Africa, there was a weekly decrease of about 48% in TB Xpert testing volumes, and the weekly number of TB positive tests fell by 33% [13]. Second, human and budgetary resources intended for managing conditions such as TB and HIV were redirected to COVID-19 testing and treatment [14]. Third, international travel restrictions and regional COVID-19 outbreaks led to temporary interruptions in supply chains for drugs, vaccines, and other health commodities [15]. Fourth, the economic consequences of COVID-19 increased financial barriers at the household and national levels to maintain routine health services [16]. In the short term, health budgets have been threatened or reduced in a number of cases, but the COVID-19 crisis also represents an opportunity to catalyze major long-term UHC financing reforms in many countries.

Evidence on the disruption of essential services has mostly come from surveys, which have shown disruptions in outpatient services, community-based care, and inpatient services [17]; HIV and TB programs [18]; and NCD diagnosis and treatment services [19]. However, there have been contextual differences in how COVID-19 has impacted health services in different LICs and LMICs. To highlight some of these differences, we have included short case studies of the impact of COVID-19 in Ghana, India, and Nigeria (S2–S4 Text).

### The potential impact of COVID-19 on grand convergence

How might this health service disruption, and the economic consequences of COVID-19, affect progress on grand convergence? Developing a probabilistic forecast of the most likely post-pandemic health trends would be incredibly challenging and fraught with uncertainty since the pandemic and its effects are still evolving. In addition, specific experiences at country and regional levels will vary from the global average, depending on the local impact of (1) the level of disruption to health services; (2) COVID-19 containment policies; (3) the application of new technologies (e.g., COVID-19 vaccines); (4) the country’s economic recovery; and (5) the political commitment to public sector spending on health, which could allow many countries to get back on track or even exceed pre-pandemic progress [20]. For example, the Severe Acute Respiratory Syndrome (SARS) epidemic in China sparked political changes that reinvigorated public finance of healthcare and the country’s move toward UHC [20].

In light of the challenges in developing long-term forecasts, we instead did an exploratory modeling analysis using stylized perturbations in historical (2009 to 2019) trends in global TB and HIV mortality rates, U5MR, and MMR, reflecting a plausible range of pandemic severity and duration. This modeling should be seen as a “what-if” exercise rather than a statement of the most likely future path. The objective of our modeling exercise was to understand, quantitatively, how much the pandemic might throw the world off track for grand convergence and what sort of acceleration in progress would be needed to get back on track. We first projected what would have happened under a “no COVID” scenario (i.e., continuation of mortality levels and trends observed over 2010 to 2019) and then under 3 different disruption scenarios (minor, moderate, and severe). We used existing evidence to develop the parameters for these 3 scenarios [21–24]. All 3 assumed a 5% increase in mortality for TB and HIV, U5MR, and MMR in 2020 and 2021 (we call this the “affected phase”). For the post COVID-19 period (from 2022 onward), our minor, moderate, and severe scenarios employ different assumptions regarding the average annual rate of change (AARC) in all-ages mortality rates (Box 1).

**Box 1. Four modeled scenarios reflecting severity of impact of COVID-19 on long-term mortality trends**

- **Base case (“no COVID-19”) scenario (S0):** In our base case, we modeled mortality rate changes for the conditions of interest (HIV, TB, under-5 mortality, and maternal mortality), assuming the pre-COVID-19 trends continue into the future. We use the AARC in mortality at the global level between 2009 and 2019 (the “historical AARC”) to estimate mortality to 2035.
- **Minor impact scenario, with accelerated progress (S1):** In this scenario, we assume that the mortality rate for the conditions of interest will increase due to COVID-19 by 5% in 2020 and 2021 during the affected phase, then decline at the historical AARC (the AARC from 2009 to 2019) for 3 years (the “recovery phase”). After that, from 2025 to 2035, we assume that the mortality rate declines at the 90th percentile of the historical AARC of LICs and LMICs (an “acceleration phase,” seen in S1 only). The 90th percentile, shown in S1 Table, is an ambitious but feasible rate of decline (many LICs and LMICs have achieved this rate or an even faster rate).
- **Moderate impact scenario, with continuation of historical trends (S2):** In this scenario, we assume that the mortality rate for the conditions of interest will increase due to COVID-19 by 5% in 2020 and 2021 during the affected phase, remain flat for 3 years (recovery phase), and then decline at the historical AARC from 2025 to 2035.
- **Severe impact scenario, with deceleration in progress (S3):** In this scenario, we assume that the mortality rate for the conditions of interest will increase due to COVID-19 by 5% in 2020 and 2021 during the affected phase, remain flat for 5 years (an extended recovery phase), and then decline at the 40th percentile of the historical AARC of LICs and LMICs from 2027 to 2035 (see S1 Table).

**Fig 1:**
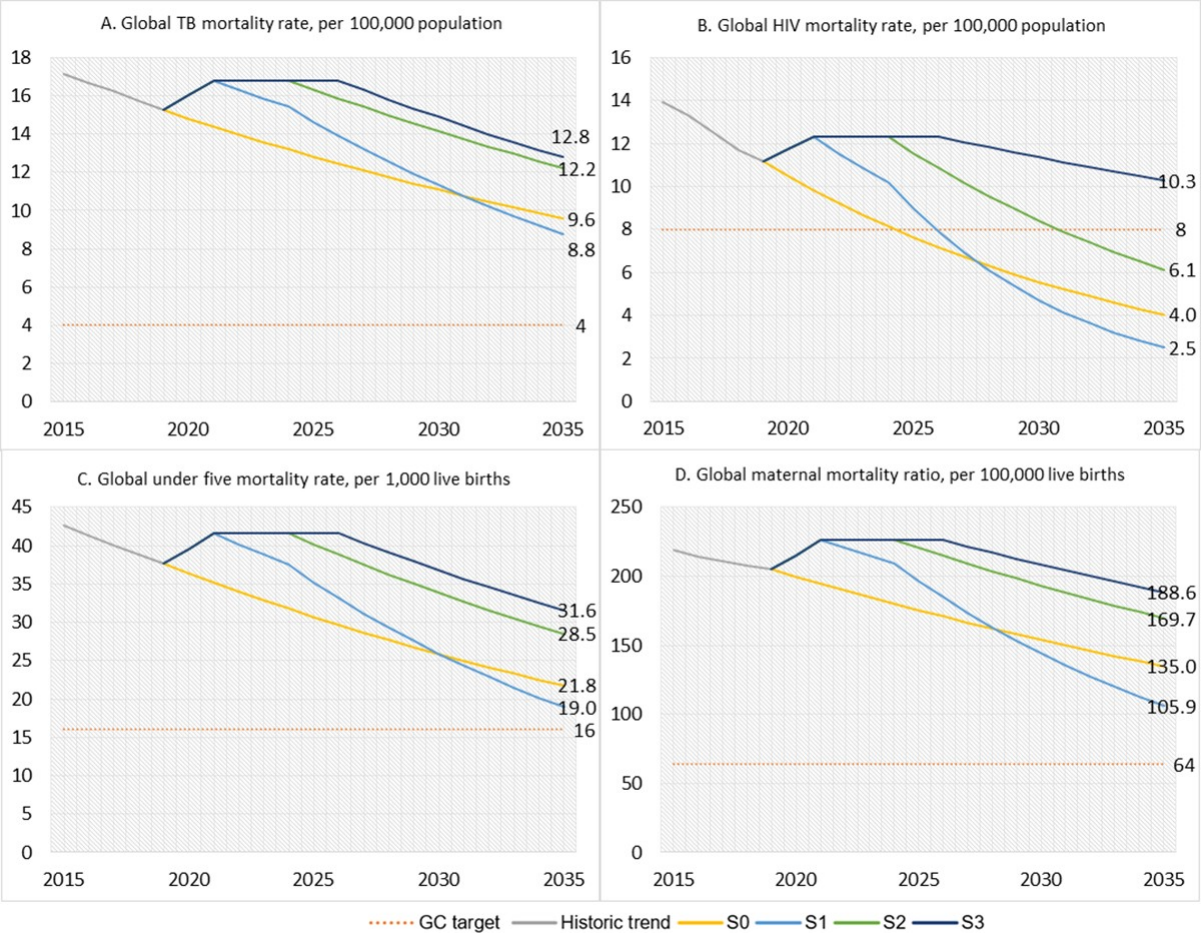
Projected global HIV, TB, and U5MRs and the global MMR under 4 different scenarios (S0–S3). Data sources that we used to estimate historical mortality rates from 2000 to 2019 included all age mortality rates for HIV and TB from the Institute for Health Metrics and Evaluation’s Global Burden of Disease Study (2000–2019) by country and year (http://ghdx.healthdata.org/gbd-results-tool); U5MR from 2000 to 2019 from the World Bank by country and year (https://data.worldbank.org/indicator/SH.DYN.MORT); MMR from 2000 to 2017 from the World Bank by country and year (https://data.worldbank.org/indicator/SH.STA.MMRT); and population estimates from the United Nations Population Division (https://population.un.org/wpp/). GC, grand convergence; MMR, maternal mortality ratio; TB, tuberculosis; U5MR, under-5 mortality rate.

Fig 1 shows the summary results of our analysis, reported at the global level. The figure shows the historical trends in the TB and HIV mortality rates, U5MR, and MMR, the trends that would be seen under the 4 scenarios S0, S1, S2, and S3, and the grand convergence targets. S2 Table shows our analysis disaggregated into LICs and LMICs. We also calculated the AARCs needed to achieve each of the 4 targets and compared these values to historical data from best-performing countries to assess feasibility.

#### TB mortality rate

Our analysis shows just how challenging it will be to achieve the grand convergence target:

- **S0 scenario:** Based on projecting forward the historical AARC (2009 to 2019) from 2020 onward, the global TB mortality rate would fall to around 9.6 per 100,000 population by 2035. If this trend were to continue beyond 2035, the grand convergence target (4 deaths per 100,000) would not be reached until 2066.
- **S1 scenario:** During the acceleration phase (2025 to 2035), the TB mortality rate would fall by 5% annually (the AARC seen at the 90th percentile of LICs and LMICs from 2009 to 2019). At this rate of decline, the TB mortality rate in 2035 would be 8.8 per 100,000, slightly lower than the S0 projection. If this rate of decline continued beyond 2035, the grand convergence target would not be reached until 2051. To achieve the grand convergence target by 2035, the AARC would need to be 10% from 2022 to 2035. Is this feasible? Historically, from 2009 to 2019, the AARC in TB mortality was only 3% globally, and the fastest AARC in TB mortality was achieved by the Republic of Moldova (AARC of 8%), followed by Angola (7%) [25]. Thus, even under the most optimistic scenario (S1), we believe that new technologies and strong political commitment will be needed in order to create an “enabling environment” for high-burden countries to achieve more rapid rates of decline in TB mortality to reach convergence. As mentioned, China increased public sector spending on health after the 2003 SARS endemic [26], which benefited its overall health system, including strengthening its immunization and TB programs. India, one of the high-burden countries for TB, aims to eliminate TB by 2025, which is likely impossible [27]. To achieve the grand convergence target for TB by 2035 will need additional political commitments for LICs and LMICs to prioritize public sector financing for health after the pandemic.
- **S2 scenario:** In this scenario, the TB mortality rate from 2025 to 2035 falls by 3% (its historical AARC from 2009 to 2019), and, at this rate of decline, the TB mortality rate in 2035 would be 12.2 per 100,000. If the same trend continued beyond 2035, the grand convergence target would not be reached until 2074.
- **S3 scenario:** Here, the TB mortality rate from 2027 to 2035 falls by 3% (the AARC seen at the 40th percentile of LICs and LMICs from 2009 to 2019), and, at this rate of decline, the TB mortality rate in 2035 would be 12.8 per 100,000. If the same trend continued beyond 2035, the grand convergence target would not be reached until after 2100.

Clearly, the original grand convergence modeling in Global Health 2035 was predicated on an epidemiological context that was more favorable than has been observed recently [1]. For example, the 2019 Lancet Commission on Tuberculosis showed how TB has become more challenging to control in several key countries over the past decade [28], trends which were not yet appreciated in the original CIH modeling [29].

#### HIV mortality rate

For HIV, there is a “good news” story to tell. Thanks to the rapid progress made in the past decade, the picture is very optimistic for the global HIV mortality rate:

- **S0 scenario:** The HIV grand convergence target (8 deaths per 100,000 population) would be reached by 2025, 10 years early (this finding is similar to what the CIH found in its Alma-Ata at 40 study).
- **S1 and S2 scenarios:** The target would also be reached early: by 2026 for S1 and by 2031 for S2.
- **S3 scenario:** In this scenario, in which the HIV mortality rate from 2027 to 2035 falls by only 2% (the AARC seen at the 40th percentile of LICs and LMICs from 2009 to 2019), the HIV mortality rate by 2035 would be 10.3 per 100,000. If this trend continued beyond 2035, the grand convergence target would not be reached until 2048.

#### Under-5 mortality rate

The picture is less optimistic for the U5MR than it is for HIV:

- **S0 scenario:** By 2035, the U5MR would fall to 21.8 per 1,000 live births, higher than the grand convergence target of 16 per 1,000 live births. The target would be reached around 2045, 10 years after the deadline. In its Alma-Ata at 40 study, the CIH projected that the target would be reached by 2038, based on the historical AARC from 2010 to 2016; our more pessimistic finding reflects the fact that the historical AARC from 2009 to 2019 is lower than that from 2010 to 2016 (the AARC from 2016 to 2019 was lower than expected).
- **S1 scenario:** During the acceleration phase (2025 to 2035), the U5MR would fall by 6% annually (the AARC seen at the 90th percentile of LICs and LMICs from 2009 to 2019). At this rate of decline, the U5MR in 2035 would be 19.0 per 1,000 live births, lower than the S0 projection. To reach the grand convergence target by 2035, an AARC of 7% would be needed between 2022 and 2035. Is this feasible? Historically, from 2009 to 2019, the AARC in the U5MR was just over 3% globally and the best-performing LICs or LMICs were Malawi, Mongolia, Uzbekistan, and Rwanda, which achieved an AARC of around 7% [30]. Thus, political commitment, technological breakthroughs, and/or more efficient scale-up of existing tools would be needed to reach convergence by 2035.
- **S2 scenario:** In this scenario, the U5MR from 2025 to 2035 falls at 3% annually (the historical AARC), and U5MR in 2035 would be 28.5 per 100,000. If this trend continued, the grand convergence target would not be achieved until 2052.
- **S3 scenario:** Here, the U5MR from 2027 to 2035 falls by 3% (the AARC seen at the 40th percentile of LICs and LMICs from 2009 to 2019), and the U5MR in 2035 would be 31.6 per 1, 000 live births. If this trend continued, the grand convergence target would not be achieved until 2058.

#### Maternal mortality ratio

For the MMR, it will also be challenging to reach the grand convergence target by 2035:

- **S0 scenario:** By 2035, the MMR would fall to 135 per 100,000 live births, much higher than the grand convergence target of 64 per 100,000 live births. Again, this finding is more pessimistic than the finding of the Alma-Ata at 40 study.
- **S1 scenario:** During the acceleration phase (2025 to 2035), the MMR would fall by 6% annually (the AARC seen at the 90th percentile of LICs and LMICs from 2009 to 2019). At this rate of decline, the MMR in 2035 would be 105.9 per 100,000, lower than the S0 projection but still much higher than the grand convergence target of 64 per 100,000. To reach the grand convergence target by 2035, an AARC of 10% would be needed between 2022 and 2035. Is this feasible? Historically, from 2009 to 2019, the AARC in the MMR was under 3% globally, and the best-performing nations were Timor-Leste and the West Bank, which achieved an AARC of around 7% [31]. As with TB and child mortality, political commitment, technological breakthroughs, and/or more efficient scale-up of existing tools would be needed to reach convergence by 2035.
- **S2 scenario:** Here, the MMR from 2025 to 2035 falls at 3% annually (the historical AARC), and the MMR in 2035 would be 169.7 per 100,000. If this trend continued, the grand convergence target would not be achieved until 2073.
- **S3 scenario:** Here, the MMR from 2027 to 2035 falls by just 2% (the AARC seen at the 40th percentile of LICs and LMICs from 2009 to 2019), and the MMR in 2035 would be 188.6 per 100,000. If this trend continued, the grand convergence target would not be achieved until 2089.

### The economic impact of COVID-19: What it means for financing UHC

Global Health 2035 argued that the grand convergence agenda could—and should—be financed primarily through domestic resources. In all LICs and LMICs, the principal means of increasing domestic health spending is greater economic growth, which, in turn, leads to larger contributions to taxation-based and social insurance–based financing systems. As their economies grow, governments also have the option to increase health spending faster than the rate of growth in general government expenditure (in LICs and LMICs, the share of GDP devoted to government health expenditure has increased by about 0.75% annually since 2000 [32]). The CIH analyses have found that these 2 mechanisms—economic growth and health budget reprioritization—would be sufficient to finance grand convergence–related interventions in LMICs. Most LICs would require external assistance to raise sufficient resources to finance grand convergence [8].

As noted previously, the growth prospects in LICs and LMICs have worsened since the publication of Global Health 2035. In October 2020, the IMF released projections of GDP growth over the next several years, factoring in countries’ COVID-19 recovery potential [33]. Most economies are expected to rebound fairly quickly as long as the pandemic is brought under control in 2021. However, since economies are expected to contract substantially in 2020, maintaining historical rates of growth over 2020 to 2035 would result in lower spending on health by 2035 relative to what could have been achieved had there not been a pandemic. For example, an LIC that has historically achieved 4% GDP growth annually would increase its GDP by 87% in 2035 relative to 2019. If that same country’s economy contracted by 5% in 2020 and remained stagnant in 2021, then resumed its 4% annual growth rate, it would only be able to increase its GDP by 65% in 2035 relative to 2019. Lesser growth in GDP by 2035 would result in less money for health, all else equal.

We note that while economic growth is a major driver of public sector spending on health, the relationship is not always a linear process, and, throughout history, there have been step changes in public financing that have often followed major crises. We previously mentioned China after SARS; other recent examples include Rwanda post-genocide and Thailand after the Asian financial crisis. The COVID-19 pandemic might provide additional motivation to increase public sector health spending in nations that have historically underspent (such as India and Nigeria).

We revisited the analyses done in the first 2 CIH reports through the lens of the pandemic and the IMF’s most recent projections of economic growth in LICs and LMICs. The starting point for our analysis was a list of 120 non-COVID-19 health interventions that would form a model essential health intervention package implemented through a UHC system [34]. As described by Blanchet and colleagues, these 120 interventions were adapted from the 3rd edition of Disease Control Priorities (DCP3) and underwent additional scrutiny as to the feasibility of their delivery amidst COVID-19–related disruptions.

We used the DCP cost model to generate estimates of the annual per capita cost of this package of 120 health interventions across 32 LICs and 47 LMICs (categorized as in the previous CIH reports) [26]. We then combined these cost estimates with population projections from the 2019 World Population Prospects to estimate the cost of the package in each country by 2035, assuming 95% coverage [35]. Previous modeling studies showed that this level of coverage would be required for these interventions to ensure the grand convergence, and related Sustainable Development Goal (SDG) targets would be achieved [36]. We then projected total domestic resources available for health by triangulating GDP growth projections from the IMF, estimates of the share of GDP devoted to public sector spending on health from WHO, and projection methods and assumptions detailed in previous CIH analyses [37].

Table 1 summarizes the findings of our analysis. By 2035, LICs would only be able to finance about a third of the costs of the package through domestic sources unless they assign substantially higher priority to health than now seems likely. LMICs would only be able to finance a little less than half. With either income group, there would inevitably be some countries with stronger growth that would be able to fully finance the package, whereas the costs would probably be out of reach for other countries. A detailed analysis of country-by-country costs was outside the scope of this report.

**Table 1:** Cost and affordability of essential non-COVID-19 health interventions in 2035.

	LICs	LMICs
Cost per capita of intervention package in 2020^a^	US$52	US$89
Projected government spending on health per capita in 2035 (2019 US$)^b^		
Scenario 1: constant share of spending vs. GDP^c^	US$15	US$48
Scenario 2: 1% annual increase in share of spending vs. GDP^d^	US$18	US$56
Annual % increase needed to finance package by 2035^e^	8.5%	5.0%

^a^Costs are adapted from the DCP cost model and converted to constant 2019 US$. We assume that labor costs grow in proportion to GDP per capita but that other costs remain constant.

^b^The quantity of interest here is GGHE-D as reported in the National Health Accounts data published by WHO. Projections combine most recent data from IMF, World Bank, and WHO and follow the methods and assumptions used by Watkins and colleagues [35].

^c^This scenario assumes that the ratio of GGHE-D to GDP remains constant over 2020–2035.

^d^This scenario assumes that the ratio of GGHE-D to GDP increases by 1% annually over 2020–2035.

^e^This number reflects the annual rate of increase in the ratio of GGHE-D to GDP that would be required to fully finance the intervention package at 95% population coverage by 2035.

DCP, Disease Control Priorities; GDP, gross domestic product; GGHE-D, general government health expenditure from domestic resources; IMF, International Monetary Fund; LIC, low-income country; LMIC, lower middle-income country.

Greater prioritization of health within government budgets could make the package affordable for the average LIC or LMIC. If the average LIC or LMIC increased its share of GDP devoted to public sector spending on health by 8.5% and 5.0% annually, respectively, it could afford the package by 2035. Although WHO’s health accounts database shows that most countries have not achieved this rate of growth in health spending historically, several countries have. Over 2000 to 2010, Rwanda, Ghana, and Togo increased the share of GDP devoted to health by over 10% annually. Across all LICs and LMICs, the 90th percentile country increased health spending by 5.1% annually over 2000 to 2018 [28].

Our assessment of the affordability of an essential health intervention package is somewhat more pessimistic than the analysis featured in Global Health 2035, which focused only on grand convergence interventions (which comprise about half of the costs of the package featured in Table 1). Our findings are broadly similar to those of Alma-Ata at 40 years, whose primary healthcare (PHC) package included interventions that addressed both grand convergence conditions as well as NCDs and injuries.

One implication of the 3 CIH analyses, taken together, is that as LICs and LMICs develop their health system responses to epidemiological and demographic transitions, they will face increasingly difficult trade-offs as to which types of interventions they can justify financing through UHC systems. Development partners continue to focus on support for grand convergence conditions—rightly so, for equity reasons. However, without a balanced approach to UHC that includes building capacity to address NCDs and injuries, countries may find themselves further behind on population health indicators as the residual burden of grand convergence conditions is addressed. Indeed, as shown in Alma-Ata at 40 years, age-specific death rates for NCDs and injuries are increasing, rather than decreasing in some LICs and LMICs (as compared to HICs, where they continue to decrease). These trends suggest that health inequalities across countries could become even more pronounced beyond 2035 if national governments and development partners focus solely on financing and implementing grand convergence–related interventions.

## Policy implications of our findings

Even before COVID-19, in its 2018 Alma-Ata at 40 report, the CIH found that if global trends in mortality achieved in 2010 to 2016 were to continue, the grand convergence targets for TB and MMR would not be reached by 2035 [8]. The CIH concluded that a massive scale-up of proven TB and maternal health interventions in poor-performing countries and the development of new health technologies would be needed to get on track. Our analysis suggests that COVID-19 will now make it extremely difficult to achieve the TB and MMR targets. And, while the 2018 report found that the 2035 targets for HIV and U5MR were feasible, which is still the case for HIV even in the face of the COVID-19 pandemic, it will now also be very challenging for countries to reach the U5MR target by 2035.

Beyond grand convergence, COVID-19 threatens much of the health-related SDGs agenda. While grand convergence conditions and interventions are the cornerstone of pro-poor UHC, it is increasingly clear that achievement of a broad-based definition of UHC that also includes prevention and essential care of NCDs and injuries is also under threat. Services for NCDs and injuries appear to be even more disrupted than services for infections and maternal and child health conditions. The pandemic is expected to adversely affect population mental health—thus, more needs to be done within and outside the health sector to respond to expected rises in the global burden of depression, anxiety, and post-traumatic stress disorder. These conditions can themselves hinder patient engagement with the health system and uptake of grand convergence interventions.

Reaching grand convergence and UHC will clearly require a major commitment to keep health at the top of the domestic and global development agendas, not just during the pandemic but also in the post-pandemic recovery years. We believe that the likelihood of getting back on track for reaching grand convergence will depend on 3 key factors, which we discuss further below: (i) how quickly COVID-19 vaccines can be deployed to reach high population coverage in LICs and LMICs; (ii) how much additional health financing can be mobilized from external and domestic sources; and (iii) whether countries can rapidly strengthen their health delivery systems, especially PHC systems, including through new delivery approaches (e.g., telemedicine).

### Global distribution of COVID-19 vaccines

One major obstacle to the deployment of vaccines to LICs and LMICs is “vaccine nationalism”—the hoarding of vaccine doses by rich nations through their bilateral purchase agreements with vaccine manufacturers [38]. The Canadian government has purchased enough doses to vaccinate all of its citizens 6 times over, and the United States and United Kingdom are not far behind, buying enough doses to vaccinate all their citizens 4 times over. The new COVID-19 Vaccine Global Access Facility (COVAX)—a global collaboration to provide equitable access to COVID-19 vaccines, with funding from the COVAX advanced market commitment—hopes to be able to distribute 2 billion doses to LICs and LMICs by the end of 2021. This would be enough to vaccinate about 15% to 20% of the population of each country with a 2-dose regimen. At the time of writing this, the Facility has purchased 2.7 billion doses [39] and argues that it is therefore on course to meet its 2021 targets.

Unless there is concerted action to redress this inequity, the lack of vaccine deployment to LICs and LMICs will impede their recovery from COVID-19 and their achievement of grand convergence and UHC. Rich nations need to (i) step up their commitments to COVAX, including to the COVAX advanced market commitment that is funding vaccine purchases for LICs and LMICs; (ii) donate their extra doses to COVAX; and (iii) agree to share the vaccine manufacturing know-how with LICs and LMICs. And the manufacturing of the vaccines needs to be quickly globalized, building on the example of the Serum Institute of India, which is manufacturing the University of Oxford/AstraZeneca vaccine. The institute initially gave doses to COVAX for distribution to LICs and LMICs until India’s surge required doses to be used only domestically (the institute will supply doses again to COVAX in late 2021). It increasingly looks likely that China will play a key role in providing COVID-19 vaccines directly, such as CoronaVac (made by the Chinese firm Sinovac), as well as development assistance for health (DAH) more broadly, to LICs and LMICs.

Insufficient manufacturing of COVID-19 vaccines and challenges to distribution of vaccines are 2 other bottlenecks to achieving global vaccine herd immunity. The Serum Institute of India, one of the largest vaccine manufacturers, produces 70 million doses every month, and it is expected to increase its capacity to produce 100 million doses per month from July 2021 [40]. Manufacturing in the US and other high-income nations is expected to ramp up in 2021 to produce enough doses to cover not just their own nations but to donate doses to COVAX or directly to LICs and LMICs. These countries will require easy to deliver, affordable vaccines in large quantities to cover their populations. The US has recently joined other nations in supporting an intellectual property waiver on COVID-19 vaccines, which could give the green light to LICs and LMICs to start manufacturing their own vaccines (this would also require technology transfer and support for scaling manufacturing facilities and capacity). Distribution challenges in some LICs and LMICs include insufficient health workers, the cold chain requirements, and reaching people in urban slums and hard-to-reach regions [41]. To date, licensed vaccines remain efficacious against all known variants of concern. Nevertheless, there remains the possibility that new variants could evade vaccine-induced immunity.

### Mobilization of domestic and external health financing

Both domestic health financing and DAH will play a critical role in getting countries back on track to reach grand convergence and UHC.

LICs and LMICs need to keep health spending at the top of their agendas. The pandemic and the measures to control it (e.g., stay-at-home orders) have had an economic impact on various sectors, with rising levels of unemployment, poverty, and income instability and inequality [42]. Countries are bound to face competing priorities over the next few years, and domestic resource mobilization and budgeting will need to carefully assess spending priorities to ensure that levels of public sector spending on health are not negatively impacted. The challenges will be particularly acute for countries with ongoing fiscal weaknesses, such as low tax revenues and high aid dependence and debt levels [43]. In order to ensure that domestic resources for health continue to support grand convergence and UHC, key priorities for country governments include the following:

- Prioritization of health in their budgets to ensure that health gains and progress toward UHC are not reversed: Although in the short run it may appear that the health sector is receiving large amounts of additional resources, in many countries, there are concerns that there may be substantial reprogramming of health outlays that cannot be sustained in the long run. The COVID-19 pandemic has shown, perhaps more starkly than ever, why it is so critical for nations to continue to prioritize health spending and, equally as important, to prioritize spending whose value for money (in terms of health impact or financial protection) is highest. As the experience of China after SARS has shown, pandemics can lead nations to reinvigorate their commitment to health. Countries that have been successful at curbing COVID-19 could use this opportunity to accelerate their trajectory to grand convergence and UHC. It is quite possible that other countries will follow suit—including even countries like the US that have, historically, had a lukewarm commitment to UHC [44].
- Planning and budgeting for health, which needs the careful attention of ministries of health: Such planning includes determining the most cost-effective priorities, improving spending efficiency, and allowing for flexibility in redirecting spending for emergencies, including procurement of COVID-19 vaccines [45]. Accountability and expenditure tracking mechanisms can help to improve transparency and avoid leakages of scarce resources. At a broader level, finance and planning ministries will need to step up efforts to stabilize funding in the long-term through multiyear programming and planning [46].
- Greater attention to tax policy: This will be critical to ensure sustainable revenue generation in the long run through measures such expansion of the tax base, improving tax compliance and administration, and diversifying sources of government revenues, including exploring the potential for imposing health taxes (e.g., taxes on alcohol, coal, and tobacco) [47,48]. Countries should renew their commitments to the 2015 Addis Tax Initiative and take actionable steps to enhance domestic revenues [49]. Research has shown that people are willing to pay higher taxes in exchange for health and education services that visibly improve their lives; however, this willingness usually requires strong social contract, as has been observed in countries in Southeast Asia and Latin America in particular [50].
- Investment in the right health interventions: The CIH noted that “countries in all regions and at all income levels, such as China, Ethiopia, Bangladesh, Mexico, and Thailand, have consistently made smart health investments and have helped to set global standards for the level of health that can be achieved at relatively modest cost” [8]. This conclusion still holds true in the COVID-19 era. The DCP3 model list of essential UHC interventions can provide a helpful starting point for countries to review their current investments and deliberate on health benefit package reforms that make better use of scarce resources [10].
- Engagement of the private sector, which consumes a large fraction of resources used for health in many LICs and LMICs: WHO and UN have recently called for an “all hands-on deck” approach and called for improved performance of the private sector, such as in expanding the health workforce and strengthening supply chains [51,52].

During the acute crisis phase, donors responded rapidly to country needs. The International Aid Transparency Initiative found that in the first 7 months of 2020, commitments to health increased compared to the same period in 2019, driven in particular by multilateral funders such as the World Bank [53]. However, given the economic impacts of COVID-19 on donor nations, future DAH flows could be under threat. Official development assistance (grants and concessional loans) has historically been the most stable external resource in developing countries during past crises [54]. Maintaining DAH will be critical for grand convergence and UHC during the COVID-19 crisis. Particular priorities for donors are the following:

- Funding global functions, especially strengthening national and global preparedness for the next pandemic, including developing and stockpiling medical countermeasures; accelerating the development of new health technologies for the “convergence conditions” (HIV, TB, malaria, and maternal and child health); and shoring up WHO’s core capacities. Even before COVID-19, there was a very strong case for using donor financing to make these preparatory global investments, such as in tackling antimicrobial resistance and strengthening pandemic preparedness, because of positive international externalities [55]. Strengthening surveillance capacity, response capacity, and regulatory capacity in low- and middle-income countries is a way to strengthen national health systems while simultaneously addressing global problems. These investments have global spillovers.
- Providing debt relief, including through mechanisms such as “debt to health swaps” (in which a creditor agrees to waive all or part of an outstanding debt obligation if the debtor government invests the funds instead into the health sector) [56].
- Providing DAH to (a) strengthen PHC systems in very resource constrained environments; and (b) selectively strengthen PHC in MICs for activities with high cross-border externalities, such as pandemic preparedness, control of antimicrobial resistance, and cross-border disease control (e.g., malaria elimination activities).

### Strengthening of primary healthcare delivery systems

Finally, strengthened PHC is the backbone for nations to respond to COVID-19, maintain essential non-COVID-19 services, and accelerate progress toward grand convergence and UHC in the post-COVID-19 recovery phase. The 2018 Astana Declaration that UN member states endorsed, arising from Global Conference on Primary Health Care, argued that “strengthening primary healthcare (PHC) is the most inclusive, effective and efficient approach to enhance people’s physical and mental health, as well as social well-being, and that PHC is a cornerstone of a sustainable health system for universal health coverage (UHC) and health-related Sustainable Development Goals” [57]. Allen and Dambha-Miller argued that the pandemic has revealed “areas where progress is needed most” [58]:

- improved coordination between PHC, public health, and other specialties;
- expanding access to PHC services, including for NCDs (S5 Text), including through telemedicine (S6 Text);
- deeper investment in prevention, integrated, team-based primary care, and care of long-term illnesses; and
- the removal of user fees and a guarantee of “comprehensive services without financial hardship.”

## Conclusions

The disruption to health services caused by COVID-19 and the accompanying economic impacts have made it more challenging to reach grand convergence by 2035 and achieve UHC. The 2035 targets for TB and MMR will be particularly difficult to reach unless we develop breakthrough technologies. Nevertheless, our analysis has also pointed to ways in which smart donor and domestic investments now and in the post-COVID-19 period can help to get countries back on track.

There are many reasons for optimism about an acceleration in progress after the pandemic. These include the pace of technological innovation (as seen with the development of COVID-19 vaccines, the fastest vaccines to have ever been developed); the adoption of new modes of healthcare delivery; and the way in which the pandemic has placed health investment at the very top of the global development agenda. There has been a sea change in how we do science—international collaborations were accelerated, there was unprecedented mobilization of research funds, and multicountry evaluations led to new health products being approved in record time.

Nevertheless, at the current rate of global vaccination, with only 20% of people in LICs and LMICs likely to be vaccinated by the end of 2021, the pandemic in these nations could last well into 2022 and possibly beyond, with ongoing health and economic repercussions. The most urgent and important task at hand for the global health community is to reach vaccine herd immunity in all nations, so that all can enter a phase of post-pandemic progress in health.

## Supporting information

S1 Table 2035 grand convergence targets and estimated outcomes under 4 different scenarios, LICs and LMICs. LIC, low-income country; LMIC, lower middle-income country.
(DOCX)

S2 Table AARC between 2009 and 2019 used in the estimation. AARC, average annual rate of change.
(DOCX)

S1 Text Packages of essential Interventions to achieve UHC. UHC, universal health coverage.
(DOCX)

S2 Text COVID-19 and Ghana’s path to convergence and UHC. COVID-19, Coronavirus Disease 2019; UHC, universal health coverage.
(DOCX)

S3 Text COVID-19 and India’s path to convergence and UHC. COVID-19, Coronavirus Disease 2019; UHC, universal health coverage.
(DOCX)

S4 Text COVID-19 and Nigeria’s path to convergence and UHC. COVID-19, Coronavirus Disease 2019; UHC, universal health coverage.
(DOCX)

S5 Text NCD control in PHC in the COVID-19 era. COVID-19, Coronavirus Disease 2019; NCD, noncommunicable disease; PHC, primary healthcare.
(DOCX)

S6 Text How telemedicine could transform NCD care: The example of hearing loss care. NCD, noncommunicable disease.
(DOCX)

## Abbreviations

AARC: average annual rate of change
CIH: Commission on Investing in Health
COVAX: COVID-19 Vaccine Global Access Facility
COVID-19: Coronavirus Disease 2019
DAH: development assistance for health
DCP3: 3rd edition of Disease Control Priorities
IMF: International Monetary Fund
LIC: low-income country
LMIC: lower middle-income country
MMR: maternal mortality ratio
NCD: noncommunicable disease
PHC: primary healthcare
SARS: Severe Acute Respiratory Syndrome
SDG: Sustainable Development Goal
TB: tuberculosis
U5MR: under-5 mortality rate
UHC: universal health coverage

## References

[1] Jamison DT, Summers LH, Alleyne G, KJ Arrow S, Berkley AB, et al. Global health 2035: a world converging within a generation. Lancet. 2013;382:1898–955. doi: 10.1016/S0140-6736(13)62105-4 24309475

[2] Barach PP, Fisher SD, Adams MJ, Burstein GR, Brophy PD, Kuo DZ, et al. Disruption of healthcare: Will the COVID pandemic worsen non-COVID outcomes and disease outbreaks? Prog Pediatr Cardiol. 2020 Jun 6:101254. doi: 10.1016/j.ppedcard.2020.101254 32837144 PMC7274978

[3] How to stop COVID-19 fuelling a resurgence of AIDS, tuberculosis and malaria. Nature. 2020 Aug 12. Available from: https://www.nature.com/articles/d41586-020-02334-0 10.1038/d41586-020-02334-0 32788740

[4] McQuaid CF, McCreesh N, Read JM, Sumner T, Houben RMGJ, White RG, et al. The potential impact of COVID-19-related disruption on tuberculosis burden. Eur Respir J. 2020;56:2001718. doi: 10.1183/13993003.01718-2020 32513784 PMC7278504

[5] Santoli JM, Lindley MC, DeSilva MB, Kharbanda EO, Daley MF, Galloway L, et al. Effects of the COVID-19 Pandemic on Routine Pediatric Vaccine Ordering and Administration—United States, 2020. MMWR Morb Mortal Wkly Rep. 2020;69:591–593. external icon. Available from: https://www.cdc.gov/mmwr/volumes/69/wr/mm6919e2.htm doi: 10.15585/mmwr.mm6919e2 32407298

[6] Joseph A. WHO warns millions of children at risk as Covid-19 pandemic disrupts routine vaccinations. 22 May 2020. Available from: https://www.statnews.com/2020/05/22/who-routine-childhood-vaccinations-disrupted-coronavirus/

[7] Jamison DT, Murphy SM, Sandbu ME. Why has under-5 mortality decreased at such different rates in different countries? J Health Econ. 2016;48:16–25. doi: 10.1016/j.jhealeco.2016.03.002 27046447 PMC4921600

[8] Watkins DA, Yamey G, Schäferhoff M, Adeyi L, Alleyne G, Alwan A, et al. Alma-Ata at 40 years: reflections from the Lancet Commission on Investing in Health. Lancet. 2018;392:1434–60. doi: 10.1016/S0140-6736(18)32389-4 30343859

[9] International Monetary Fund. Regional economic outlook: sub-Saharan Africa. Washington, DC: International Monetary Fund; 2017.

[10] Watkins DA, Jamison DT, Mills A, Atun R, Danforth K, Glassman A, et al. Universal Health Coverage and Essential Packages of Care. In: Disease Control Priorities. 3rd ed, Volume 9. Washington, DC: World Bank. Available from: http://dcp-3.org/chapter/2551/essential-universal-health-coverage 30212154

[11] Schäferhoff M, Chodavadia P, Martinez S, McDade KK, Fewer S, Silva S, et al. International Funding for Global Common Goods for Health: An Analysis Using the Creditor Reporting System and G-FINDER Databases. Health Syst Reform. 2019;5(40):350–65. doi: 10.1080/23288604.2019.1663646 31710516

[12] International Working Group on Financing Preparedness. From panic and neglect to investing in health security. Washington (DC): World Bank; 2017. Available from: pubdocs.worldbank.org/en/890291523304595565/FINAL-IWG-Report-3-5-18.pdf

[13] National Institute for Communicable Diseases. Division of the National Health Laboratory Service. Impact of COVID-19 intervention on TB testing in South Africa. 2020 May 10. Available from: https://www.nicd.ac.za/wp-content/uploads/2020/05/Impact-of-Covid-19-interventions-on-TB-testing-in-South-Africa-10-May-2020.pdf

[14] STOP TB Partnership. Rapid assessment: The TB response is heavily impacted by the COVID-19 pandemic. 2020 Apr. Available from: http://www.stoptb.org/news/stories/2020/ns20_014.html

[15] UNICEF. Geneva Palais briefing note on the impact of COVID-19 mitigation measures on vaccine supply and logistics. 2020 May 1. Available from: https://www.unicef.org/press-releases/geneva-palais-briefing-note-impact-covid-19-mitigation-measures-vaccine-supply-and

[16] International Monetary Fund. World Economic Outlook, October 2020: A Long and Difficult Ascent. Available from: https://www.imf.org/en/Publications/WEO/Issues/2020/09/30/world-economic-outlook-october-2020

[17] WHO. Pulse survey on continuity of essential health services during the COVID-19 pandemic interim report. 2020 Aug 27. Available from: https://www.who.int/publications/i/item/WHO-2019-nCoV-EHS_continuity-survey-2020.1

[18] Friends of the Global Fight Against AIDS, Tuberculosis and Malaria. How COVID-19 is affecting the global response to AIDS, tuberculosis and malaria. Global fund survey: majority of HIV, TB and malaria programs face disruptions as a result of covid-19. 2020 Dec 8. Available from: theglobalfight.org/covid-aids-tb-malaria/

[19] WHO. The impact of the COVID-19 pandemic on noncommunicable disease resources and services: results of a rapid assessment. 2020 May. Available from: https://www.who.int/publications/i/item/ncds-covid-rapid-assessment

[20] Huang Y. The SARS Epidemic and Its Aftermath in China: a Political Perspective. In: Knobler S, Mahmoud A, Lemon S, et al., editors. Institute of Medicine (US) Forum on Microbial Threats. Learning from SARS: Preparing for the Next Disease Outbreak: Workshop Summary. Washington (DC): National Academies Press (US); 2004. Available from: https://www.ncbi.nlm.nih.gov/books/NBK92479/22553895

[21] STOP TB Partnership. The potential impact of the covid-19 response on tuberculosis in high-burden countries: a modelling analysis. Available from: http://www.stoptb.org/assets/documents/news/Modeling%20Report_1%20May%202020_FINAL.pdf

[22] Hogan AB, Jewell BL, Sherrard-Smith E, Vesga JF, Watson OJ, Whittaker C, et al. Potential impact of the COVID-19 pandemic on HIV, tuberculosis, and malaria in low-income and middle-income countries: a modelling study. Lancet Glob Health. 2020;8(9):e1132–e41. doi: 10.1016/S2214-109X(20)30288-6 32673577 PMC7357988

[23] Jewell BL, Mudimu E, Stover J, ten Brink D, Phillips AN, Martin-Hughes R, et al. Potential effects of disruption to HIV programmes in sub-Saharan Africa caused by COVID-19: results from multiple mathematical models. Lancet HIV. 2020;7(9):e629–40. doi: 10.1016/S2352-3018(20)30211-3 32771089 PMC7482434

[24] Roberton T, Carter ED, Chou VB, Stegmuller R, Jackson BD, Tam Y, et al. Early estimates of the indirect effects of the COVID-19 pandemic on maternal and child mortality in low-income and middle-income countries: a modelling study. Lancet Glob Health. 2020 Jul 1;8(7). Available from: www.thelancet.com/journals/langlo/article/PIIS2214-109X(20)30229-1/fulltext 10.1016/S2214-109X(20)30229-1 PMC7217645 32405459

[25] GBD Compare Viz Hub. Available from: https://vizhub.healthdata.org/gbd-compare/#

[26] Bouey J. Strengthening China’s Public Health Response System: From SARS to COVID-19. Am J Public Health. 2020;110:939–940. doi: 10.2105/AJPH.2020.305654 32213081 PMC7287547

[27] Dey S. India aims to eliminate tuberculosis by 2025, five years ahead of global target. Times of India. 2019 Sep 25. Available from: http://timesofindia.indiatimes.com/articleshow/71298632.cms?utm_source=contentofinterest&utm_medium=text&utm_campaign=cppst

[28] Reid MJA, Arinaminpathy N, Bloom A, Bloom BR, Boehme C, Chaisson R, et al. Building a tuberculosis-free world: The Lancet Commission on tuberculosis. Lancet. 393(10178):1331–84. doi: 10.1016/S0140-6736(19)30024-8 30904263

[29] Watkins DA, Qi J, Kawakatsu Y, Pickersgill SJ, Horton SE, Jamison DT. Resource requirements for essential universal health coverage: a modelling study based on findings from Disease Control Priorities, 3rd edition. Lancet Glob Health. 2020;8:e829–39. doi: 10.1016/S2214-109X(20)30121-2 32446348 PMC7248571

[30] The World Bank DataBank. Mortality rate, under-5 (per 1,000 live births). Available from: https://data.worldbank.org/indicator/SH.DYN.MORT

[31] The World Bank DataBank. Maternal mortality ratio (modeled estimate, per 100,000 live births). Available from: https://data.worldbank.org/indicator/SH.STA.MMRT

[32] WHO. Global Health Expenditure Database. Available from: https://apps.who.int/nha/database/ViewData/Indicators/en

[33] IMF. World Economic and Financial Surveys. World Economic Outlook Database. 2020 Oct. Available from: https://www.imf.org/en/Publications/WEO/weo-database/2020/October

[34] Blanchet K, Alwan A, Antoine C, Cros MJ, Feroz F, Guarcha TA, et al. Protecting essential health services in low-income and middle-income countries and humanitarian settings while responding to the COVID-19 pandemic. BMJ Glob Health. 2020;5:e003675. Available from: https://gh.bmj.com/content/5/10/e003675 doi: 10.1136/bmjgh-2020-003675 33028701 PMC7542611

[35] United Nations. Population Division. The 2019 Revision of World Population Prospects. Available from: Bottom of Form https://population.un.org/wpp/

[36] Boyle CF, Levin C, Hatefi A, Madriz S, Santos N. Achieving a “Grand Convergence” in Global Health: Modeling the Technical Inputs, Costs, and Impacts from 2016 to 2030. PLoS ONE. 2015;10: e0140092. doi: 10.1371/journal.pone.0140092 26452263 PMC4599920

[37] Watkins DA, Qi J, Saxenian H, Horton SE. Costing universal health coverage: an update of the DCP3 costing model for the Lancet Commission on Investing in Health. DCP3 Working Paper Series. 2018. Working Paper #24. Available from: http://dcp-3.org/resources/costing-universal-health-coverage-update-dcp3-costing-model-lancet-commission-investing

[38] McAdams D, McDade KK, Ogbuoji O, Johnson M, Dixit S, Yamey G. Incentivising wealthy nations to participate in the COVID-19 Vaccine Global Access Facility (COVAX): a game theory perspective. BMJ Glob Health. 2020;5:e003627. Available from: https://gh.bmj.com/content/5/11/e003627 doi: 10.1136/bmjgh-2020-003627 33257418 PMC7705419

[39] Launch & Scale Speedometer. Mapping covid-19 vaccine pre-purchases across the globe. December 18, 2020: weekly vaccine research update. Available from: https://launchandscalefaster.org/COVID-19

[40] Reuter. India’s Serum Institute to raise output to 100 mln AstraZeneca doses by July, not end-May. Apr 21, 2021. Available from: https://www.reuters.com/world/india/indias-serum-institute-raise-output-100-mln-astrazeneca-doses-by-july-not-end-2021-04-21/

[41] Acharya KP, Ghimire TR, Subramanya SH. Access to and equitable distribution of COVID-19 vaccine in low-income countries. NPJ Vaccines. 2021;6:54. doi: 10.1038/s41541-021-00323-6 33854072 PMC8047027

[42] Slotman JR, UN/DESA Policy Brief #86: The long-term impact of COVID-19 on poverty. 2020 Oct 5. Available from: https://www.un.org/development/desa/dpad/publication/un-desa-policy-brief-86-the-long-term-impact-of-covid-19-on-poverty/

[43] Kose MA, Ohnsorge F, Nagle P, Sugawara N. Caught by the cresting Debt Wave. Past debt crises can teach developing economies to cope with COVID-19 financing shocks. 2020 Jun. Available from: https://www.imf.org/external/pubs/ft/fandd/2020/06/pdf/COVID19-and-debt-in-developing-economies-kose.pdf

[44] Cousart C, Cardwell A. American Rescue Plan Could Significantly Enhance Health Insurance Coverage. National Academy for State Health Policy; 2021 Mar 1. Available from: https://www.nashp.org/american-rescue-plan-could-significantly-enhance-health-insurance-coverage/

[45] Barroy H, Margini F, Kutzin J, Ravishankar N, Piatti- Fünfkirchen M, Gurazada S, et al. If you’re not ready, you need to adapt: lessons for managing public finances from the COVID-19 response. Available from: https://p4h.world/en/blog-lessons-for-managing-public-finances-from-COVID-19-response

[46] OECD. Building a coherent response for a sustainable post-COVID-19 recovery: Towards a Policy Coherence Roadmap. 2020 Jul 13. Available from: http://www.oecd.org/governance/pcsd/PRELIMINARY%20VERSION_PCSD_Policy-Response-Covid19_13%20July%202020.pdf

[47] IMF. Special Series on COVID-19: Tax Policy for Inclusive Growth after the Pandemic. 2020 Dec 16. Available from: https://www.imf.org/en/Publications/SPROLLs/covid19-special-notes

[48] Mullins P, Gupta S, Liu J. Domestic Revenue Mobilization in Low Income Countries: Where To From Here? 2020 Dec 10. Available from: https://www.cgdev.org/publication/domestic-revenue-mobilization-low-income-countries-where-here

[49] Financing for Development Conference. The Addis Tax Initiative–Declaration. Available from: https://www.addistaxinitiative.net/sites/default/files/resources/ATI-Declaration-EN.pdf

[50] World Bank Group and PricewaterhouseCoopers. Paying taxes, the global picture. Available from: http://documents1.worldbank.org/curated/pt/427711468140953709/pdf/379620DB1Paying1Taxes.pdf

[51] United Nations. Department of Ecnomic and Social Affairs. Private sector leaders commit to mobilizing resources to build back better from COVID-19. Available from: https://www.un.org/development/desa/financing/post-news/private-sector-leaders-commit-mobilizing-resources-build-back-better-covid-19

[52] Clarke D, Hellowell M, O’Hanlon B, Eldridge C, Impact for Health. All hands on deck: mobilising the private sector for the COVID-19 response. 2020 Apr 6. Available from: https://hsgovcollab.org/en/news/all-hands-deck-mobilising-private-sector-covid-19-response

[53] Tracking aid flows in light of the Covid-19 crisis. 2020 Aug 11. Available from: https://reliefweb.int/report/world/tracking-aid-flows-light-covid-19-crisis

[54] OECD. "Six decades of ODA: insights and outlook in the COVID-19 crisis", in Development Co-operation Profiles. Paris: OECD Publishing. doi: 10.1787/5e331623-en

[55] Yamey G, Jamison D, Hanssen O, Soucat A. Financing global common goods for health: when the world is a country. Health Syst Reform. 2019;5(4):334–49. doi: 10.1080/23288604.2019.1663118 31860402

[56] Wuennenberg L. Debt-to-health swaps: financing health system resilience beyond the covid-19 pandemic. International Institute for Sustainable Development. Sustainable Recovery 2020. 2020 Jun 12. Available from: https://www.iisd.org/sustainable-recovery/debt-to-health-swaps-financing-health-system-resilience-beyond-the-covid-19-pandemic/

[57] Global Conference on Primary Care. Declaration of Astana. Astana, Kazakhstan: Oct 25–26, 2018. Available from: https://www.who.int/docs/default-source/primary-health/declaration/gcphc-declaration.pdf

[58] Allen LN, Dambha-Miller H. COVID-19 and international primary care systems: Rebuilding a stronger primary care. BJGP Open. 2020. doi: 10.3399/bjgpopen20X101130 Available from: https://bjgpopen.org/content/bjgpoa/early/2020/09/09/bjgpopen20X101130.full.pdf 32900706 PMC7606142

