# Supplementary material for: Achieving global mortality reduction targets and universal health coverage: The impact of COVID-19
Source: PLoS Med. 2021 Jun 24;18(6):e1003675. doi: 10.1371/journal.pmed.1003675 (PMC8270396; doi:10.1371/journal.pmed.1003675)
Supplement: S1 Table — LIC, low-income country; LMIC, lower middle-income country. (DOCX) [file pmed.1003675.s001.docx]

**S1 Table. 2035 grand convergence targets and estimated outcomes under four different scenarios, low-income countries and lower middle-income countries**

| **Indicator** | **2035 grand convergence target** | **Estimated outcome, S0** | | **Estimated outcome, S1** | | **Estimated outcome, S2** | | | **Estimated outcome, S3** | |
| --- | --- | --- | --- | --- | --- | --- | --- | --- | --- | --- |
|  |  | **LICs** | **LMICs** | **LICs** | **LMICs** | **LICs** | **LMICs** | **LICs** | | **LMICs** |
| **TB mortality rate (per 100 000 population)** | 4 | 21.5 | 15.0 | 21.6 | 14.2 | 28.5 | 19.4 | 32.2 | | 20.9 |
| **HIV mortality rate (per 100 000 population)** | 8 | 6.9 | 3.9 | 7.0 | 2.4 | 12.7 | 5.9 | 32.2 | | 9.8 |
| **U5MR (per 1 000 live births)** | 16 | 37.5 | 27.9 | 33.8 | 24.6 | 49.7 | 36.7 | 56.7 | | 41.0 |
| **MMR (per 100 000 live births)** | 64 | 251.7 | 168.6 | 217.1 | 132.6 | 328.0 | 212.1 | 395.3 | | 236.4 |
