# Supplementary material for: Achieving global mortality reduction targets and universal health coverage: The impact of COVID-19
Source: PLoS Med. 2021 Jun 24;18(6):e1003675. doi: 10.1371/journal.pmed.1003675 (PMC8270396; doi:10.1371/journal.pmed.1003675)
Supplement: S2 Table — AARC, average annual rate of change. (DOCX) [file pmed.1003675.s002.docx]

**S2 Table. AARC between 2009-2019 used in the estimation**

|  | **90^th^ percentile for LICs and LMICs (S1)** | **Global (S0 and S2)** | **40^th^ percentile for LICs and LMICs (S3)** | **AARC required to achieve GC (build upon S1’s assumptions of COVID-19 impact), 2022-2035** |
| --- | --- | --- | --- | --- |
| **TB mortality rate (per 100 000 population)** | 5% | 3% | 3% | 10% |
| **HIV mortality rate(per 100 000 population)** | 12% | 6% | 2% | 3% |
| **U5MR(per 1 000 live births)** | 6% | 3% | 3% | 7% |
| **MMR(per 100 000 live births)** | 6% | 3% | 2% | 10% |
