# Supplementary material for: Achieving global mortality reduction targets and universal health coverage: The impact of COVID-19
Source: PLoS Med. 2021 Jun 24;18(6):e1003675. doi: 10.1371/journal.pmed.1003675 (PMC8270396; doi:10.1371/journal.pmed.1003675)
Supplement: S1 Text — UHC, universal health coverage. (DOCX) [file pmed.1003675.s003.docx]

### **S1 Text. Packages of essential interventions to achieve UHC**

The third edition of the Disease Control Priorities Project (DCP3), which was seven years in the making and had over 500 authors worldwide, provides “a review of evidence on cost-effective interventions to address the burden of disease in low- and middle-income countries” (<http://dcp-3.org/about-project>). DCP3 included 21 distinct packages of essential interventions, each oriented to a different domain (e.g. an essential package of reproductive health interventions, an essential package of pandemic preparedness interventions). Inclusion criteria for these interventions were that they addressed a high disease burden, provided good value for money, and were feasible to deliver in LICs and MICs. From these 21 packages, DCP3 then developed an overarching package, called the essential UHC package (EUHC), comprising 218 interventions that could be delivered across five platforms (the first four of these represent primary care delivery settings):

- population-based health interventions (n=13 interventions), e.g., active case finding for TB, ensuring vaccine security at national and subnational level
- community-based health interventions (n=59), e.g., antenatal and postpartum education on family planning, school-based education on sexual health, nutrition, and health lifestyle
- health centers (n=68), e.g., screening and management of hypertensive disorders in pregnancy, provision of aspirin for all cases of suspected acute myocardial infarction
- first-level hospitals (n=58), e.g., surgery for ectopic pregnancy, management of severe malaria
- referral and specialty hospitals (n=20), e.g. repair of obstetric fistula, surgery for trachomatous trichiasis

DCP3 also defined a “highest priority package” (HPP), a sub-set of 108 interventions from the EUHC package that were tailored to the health needs and resource limitations of the poorest countries. The interventions in the HPP were those that offered the highest chance of providing financial protection (e.g. of averting catastrophic medical expenses), prioritized the poorest, and offered the best value for money.
