# Supplementary material for: Achieving global mortality reduction targets and universal health coverage: The impact of COVID-19
Source: PLoS Med. 2021 Jun 24;18(6):e1003675. doi: 10.1371/journal.pmed.1003675 (PMC8270396; doi:10.1371/journal.pmed.1003675)
Supplement: S2 Text — COVID-19, Coronavirus Disease 2019; UHC, universal health coverage. (DOCX) [file pmed.1003675.s004.docx]

### **S2 Text. COVID-19 and Ghana’s path to convergence and UHC**

Deloitte estimates that Ghana’s GDP growth could fall from a target of 6.8% to about 2.6% in 2020 due to the COVID-19 pandemic [^[[1]](#endnote-1)^], which would have a significant impact on all capital expenditures, including health. A recent survey by the Ghana Statistical Service (GSS), in collaboration with the United Nations Development Programme and the World Bank, shows that the COVID-19 pandemic has contributed to about a 72% and a 90% reduction in production and sales of local businesses, respectively [1,^[[2]](#endnote-2)^].

Low- and middle-income countries depend on large scale health programmes to control and treat people living with HIV and TB [^[[3]](#endnote-3)^]. A recent modelling study suggested that in these countries, HIV and TB related deaths over the next five years may increase by up to 10% and 20%, respectively, compared to a no COVID-19 pandemic scenario [^[[4]](#endnote-4)^] The impact of the pandemic on HIV and TB prevention and treatment services could be minimised by maintaining core services: continued access to antiretroviral medicines (ARVs), and maintenance of TB diagnosis and treatment. Unfortunately, such maintenance of services has not been achieved. The gains made in control of HIV and TB by the Global Fund and the Government of Ghana are likely to be reversed without a proper mechanism to provide essential services to affected populations.

The pandemic in Ghana has contributed to the reduction in timely diagnosis and provision of care for TB-affected households and treatment of new cases. This reduction is related to the prolonged measures that the government took to suppress transmission of SARS-CoV-2. For instance, the recent GSS survey indicated that since March 16, about 27% of children scheduled for all forms of vaccination, including BCG, missed their vaccine doses for reasons related to COVID-19 [2]. In addition, 8.6% of households could not buy medicines when they needed to, and about 6.1% of households that attempted to access medical treatment (1.3% of all households) were unsuccessful [1,2].

These data help to explain the decrease in rates of diagnosis of TB and HIV, treatment initiation, and cases management of drug-resistant TB. COVID-19 led to an unintended disruption of government spending on non-COVID health services and a partial three-week stay-at-home order (“lockdown”) that restricted movement in Accra and Greater Kumasi metropolitan areas of the country. COVID-19 also led to greater stigmatization of patients with TB (the WHO notes that “both diseases have similar symptoms such as cough, fever and difficulty breathing,” [^[[5]](#endnote-5)^] although TB has a slower onset and longer incubation period).

Access to voluntary testing for TB is likely to have been curtailed in Ghana by the pandemic. This reduced access is in part because of the redirection of human and material resources away from TB towards the fight against COVID-19, but also because the social stigma of having a cough or being unwell may keep people from seeking care, leading to more advanced disease and greater infectiousness. TB-related stigma in Ghana is well described. While such stigma predates COVID-19, it may have been worsened by the COVID-19 pandemic given the similarities in symptoms between the two diseases [^[[6]](#endnote-6)^]. People living with TB and TB survivors are likely to be at higher risk than the general population of COVID-19 and associated adverse outcomes due to chronic lung damage coupled with stigmatization [^[[7]](#endnote-7)^].

Similarly, the disruption of HIV services, including ARVs, due to high health system demand in the current COVID-19 era and the fear of contracting SARS-CoV-2 at health facilities due to impaired immune response has prevented HIV positive patients from accessing routine medication. There are reports of several hospitals running out of ARVs during the pandemic [^[[8]](#endnote-8)^]. Patients’ reduced access to ARVs and TB drugs could have led to increased viral loads. Such treatment interruption, together with reduced access to pre-exposure prophylaxis and condoms, in turn may have affected HIV transmission rates [^[[9]](#endnote-9)^].

At the same time that Ghana develops a response to its disruptions in TB and HIV care, the country also has the opportunity to reform the National Health Insurance Scheme (NHIS) more broadly. NHIS coverage is currently only about 40% and favors those employed in the formal sector; most Ghanaians are left vulnerable to financial risk from seeking healthcare (with HIV and TB services being notable exceptions). Opportunities for improved performance of NHIS post-COVID include payment reforms, enhancement of quality standards in public facilities, and other efforts to improve confidence in the scheme and increase enrolment.

1. Deloitte. Economic Impact of the Covid-19 Pandemic on the Economy of Ghana Summary of Fiscal Measures and Deloitte views. April 2020. Available at: https://www2.deloitte.com/content/dam/Deloitte/gh/Documents/about-deloitte/gh-economic-Impact-of-the-Covid-19-Pandemic-on-the-Economy-of-Ghana_06042020.pdf [↑](#endnote-ref-1)
2. Service GS. Brief on COVID-19 Households and Jobs Tracker Wave 1. Ghana Statistical Service; 2020 30/07/2020. [↑](#endnote-ref-2)
3. WHO. Global tuberculosis report 2019. 21/11/2020. Available at: <https://www.who.int/tb/publications/global_report/en>. [↑](#endnote-ref-3)
4. #### Hogan AB, Jewell BL, Sherrard-Smith E, Vesga JF, Watson OJ, Whittaker C, et al. Potential impact of the COVID-19 pandemic on HIV, tuberculosis, and malaria in low-income and middle-income countries: a modelling study. Lancet Glob Health. 2020;8(9):e1132-e41.

   [↑](#endnote-ref-4)
5. WHO. Tuberculosis and COVID-19. Available at: https://www.who.int/teams/global-tuberculosis-programme/covid-19 [↑](#endnote-ref-5)
6. Datta S, Evans CA. Healthy survival after tuberculosis. Lancet Infect Dis. 2019;19(10):1045-7. DOI:<https://doi.org/10.1016/S1473-3099(19)30387-1> [↑](#endnote-ref-6)
7. Enyogoi F, Kibet JK, Wadunde I, Maina JK. Covid-19 Risk Stratification: Community Paramedicine Health care (CPH) Model for handling Cancer, HIV/AIDS, and Diabetic Patients during the Coronavirus Pandemic. IJAED. 2020;2(1).  [↑](#endnote-ref-7)
8. # Modern Ghana. HIV antiretroviral drug runs out; 3 patients compelled to share one bottle to survive.2020. 5. Available at: <https://www.modernghana.com/news/1047980/hiv-antiretroviral-drug-runs-out-3-patients-compe.html>

   [↑](#endnote-ref-8)
9. Jewell BL, Mudimu E, Stover J, ten Brink D, Phillips AN, Martin-Hughes R, et al. Potential effects of disruption to HIV programmes in sub-Saharan Africa caused by COVID-19: results from multiple mathematical models. Lancet HIV: [volume 7, issue 9](https://www.thelancet.com/journals/lanhiv/issue/vol7no9/PIIS2352-3018(20)X0009-9), e629-e640, September 01, 2020. DOI:<https://doi.org/10.1016/S2352-3018(20)30211-3>. [↑](#endnote-ref-9)
