# Supplementary material for: Achieving global mortality reduction targets and universal health coverage: The impact of COVID-19
Source: PLoS Med. 2021 Jun 24;18(6):e1003675. doi: 10.1371/journal.pmed.1003675 (PMC8270396; doi:10.1371/journal.pmed.1003675)
Supplement: S3 Text — COVID-19, Coronavirus Disease 2019; UHC, universal health coverage. (DOCX) [file pmed.1003675.s005.docx]

### **S3 Text. COVID-19 and India’s path to convergence and UHC**

Since 2005, total health expenditure (THE) has accounted for less than 3.8% of India’s GDP, and almost two thirds of THE was paid by out-of-pocket (OOP) expenditures. In the year 2016, government health expenditure was only 1.17% of the GDP, a share that is even lower than the average for low-income countries (the average is 1.57%). Public spending on healthcare has remained at one percent for the past one decade, despite two major policy initiatives in the past one and half decades, the National Health Mission (NHM) since 2005 and Ayushman Bharat since 2018. India’s response to COVID-19 was upon this background of low spending on health.

On March 25 2020, India instituted one of the strictest and longest lockdowns in the world in an attempt to curb the spread of the COVID and to simultaneously buy time to shore up its public health resources to combat the pandemic. One fallout of the lockdown has been disruption to non-COVID healthcare services, particularly TB services. India accounts for a quarter of all cases of TB and multidrug-resistant TB (MDR-TB) [^[[1]](#endnote-1)^]. To examine the potential impact of short-term lockdowns on TB incidence and mortality over the next 5 years (2020-2025) in high-burden countries, the Stop TB Partnership in collaboration with the Imperial College, Avenir Health, Johns Hopkins University and USAID, conducted a modeling analysis. The model predicted that globally, a 3-month lockdown and a 10-month restoration of TB services would lead to 6.3 million TB cases and 1.4 million deaths due to TB. In India, during this period, a 3-month lockdown and 10-month recovery would lead to an increase of 1.8 million cases (12%) and 510,00 TB deaths (19%) [^[[2]](#endnote-2)^]. A rapid national survey of TB program officers found a roughly 80% decline in daily TB notifications during the lockdown period compared to the average daily notifications in previous years [2, ^[[3]](#endnote-3)^].

India’s Nikshay (End TB) portal, the “web enabled patient management system for TB control under the National Tuberculosis Elimination Programme,” reported a decline in the number of patients notified by the private and public sectors during the lockdown period compared to the number of notified cases in the preceding two years) [^[[4]](#endnote-4)^]. In 2020, only 58% cases were notified by the public sector and 45% by the private sector, with an overall notification rate of only 53% (public and private sector combined) as compared to 84% in 2019 (more than a 30% drop in overall notifications) [^[[5]](#endnote-5)^]. Given the predominance of private delivery in the treatment of TB in India, we analyzed consumption of TB drugs. Using PHARMATRAC data, a market-based database that captures retail sales, we see a decline in sales volume and value for TB drugs for the period January 2020 to June 2020 (Fig A). We see a decline of 14.0% in sales volume of all five anti TB drugs (ethambutol, pyrazinamide, rifampicin, streptomycin and isoniazid) from the period January 2020 to May 2020, as compared to a decline of 1.7% from January to May 2019, suggesting that the COVID-19 pandemic has impacted access to TB services.


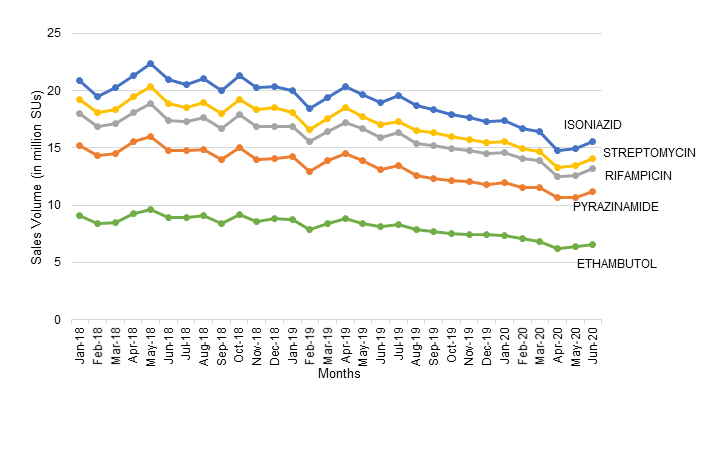


**Fig A. Sales volume (in million SUs) of TB drugs (plain) from Jan 2018 -Jun 2020**

Overall, the COVID-19 pandemic and the accompanying lockdown has resulted in severe disruptions of TB treatment services in the country in both the public and the private sector. Innovative strategies will be required to accelerate the detection of new cases and to support patients on treatment.

1. Husain AA, Monaghan TM, and Kashyap RS. Impact of COVID-19 pandemic on tuberculosis care in India. August 2020. Available at: <https://www.clinicalmicrobiologyandinfection.com/article/S1198-743X(20)30496-1/fulltext> [↑](#endnote-ref-1)
2. STOP TB Partnership: The potential impact of the covid-19 response on tuberculosis in high-burden countries: a modelling analysis. Available at: http://www.stoptb.org/assets/documents/news/Modeling%20Report_1%20May%202020_FINAL.pdf [↑](#endnote-ref-2)
3. STOP TB Partnership: April 2020. [Rapid assessment: The TB response is heavily impacted by the COVID-19 pandemic.](http://www.stoptb.org/news/stories/2020/ns20_014.html) Available at: http://www.stoptb.org/news/stories/2020/ns20_014.html [↑](#endnote-ref-3)
4. Tracking aid flows in light of the Covid-19 crisis. 11 Aug 2020. Available at: <https://reliefweb.int/report/world/tracking-aid-flows-light-covid-19-crisis> [↑](#endnote-ref-4)
5. Nikshay Reports. Available at: <https://reports.nikshay.in/Reports/TBNotification> [↑](#endnote-ref-5)
