# Supplementary material for: Achieving global mortality reduction targets and universal health coverage: The impact of COVID-19
Source: PLoS Med. 2021 Jun 24;18(6):e1003675. doi: 10.1371/journal.pmed.1003675 (PMC8270396; doi:10.1371/journal.pmed.1003675)
Supplement: S4 Text — COVID-19, Coronavirus Disease 2019; UHC, universal health coverage. (DOCX) [file pmed.1003675.s006.docx]

### **S4 Text. COVID-19 and Nigeria’s path to convergence and UHC**

As of December 28, 2020, the Nigeria Centre for Disease Control has confirmed 84,412 cases of COVID-19 and 1,254 deaths from the disease [^[[1]](#endnote-1)^]. The COVID-19 pandemic has also affected the country’s macroeconomy, its fiscal space for health, service use, and catastrophic expenditure on health. Nigeria currently spends less than 5% of its federal budget on health. Despite its COVID-19 pandemic, Nigeria cut funding for local, primary healthcare services by more than 40% this year, which could affect immunizations, childcare, maternal healthcare and family planning services [^[[2]](#endnote-2)^].

Nigeria’s economy is largely dependent on crude oil revenue, accounting for about 65% of economic activity. Significant reductions in international trade and travel due to the COVID-19 pandemic contributed to the reduction in crude oil prices [^[[3]](#endnote-3)^] (to their lowest in the last 18 years). This reduction has implications for Nigeria’s overall revenue/gross domestic product, which contracted by 3% in the second quarter of 2020 [^[[4]](#endnote-4)^], which in turn reduced the overall resources available for health and other social sectors. Despite this economic contraction, the Nigerian government has prioritized available resources to focus more on the COVID-19 pandemic (evidenced by the amended 2020 national budget), which may divert already constrained resources away from other health system issues.

Health programs in Nigeria that have major funding from donors, such as routine immunization and HIV programs, are also likely to be affected by the predicted reductions in development aid. Development Initiatives estimates that in 2020 total official development assistance could fall by US$ 25 billion from its 2019 levels as a result of economic recession in donor nations [^[[5]](#endnote-5)^]. It will be important to prioritize aid effectiveness including resource tracking and accountability in the coming years.

The COVID-19 pandemic has disrupted service availability and use of health services, including for noncommunicable diseases, across the globe [^[[6]](#endnote-6)^]. These are consequences of reprioritization of staff to respond to the COVID emergency, limited personal protective equipment for health workers, and reduced demand for services due to lock down restrictions and the stigma and fear of COVID-19 infections. Analysis of data from Nigeria’s District Health Information System 2 suggests that the uptake of routine immunization and maternal health services have been adversely affected since the onset of the pandemic. Measles vaccination and antenatal attendance have seen an almost 10% decrease since the onset of the COVID-19 pandemic. Despite the downside of the effects of COVID-19 in Nigeria, the pandemic has also highlighted potential opportunities including the role of digital health (see Box 6), which can be leveraged to support the development of resilient health systems.

Even before the COVID-19 pandemic, estimates suggested that at least 40% of the population were living in poverty [^[[7]](#endnote-7)^]. Also, Nigeria has one of the highest out-of-pocket expenditures on health (as a proportion of total health expenditure) in Africa, further pushing the poor and vulnerable population into impoverishment due to catastrophic spending on health [^[[8]](#endnote-8)^].

With the onset of the COVID-19 pandemic, employment rates fell significantly. A study by the National Bureau of Statistics found that of the surveyed population, almost 42% lost their employment and household income fell in almost 80% of households due to the pandemic [^[[9]](#endnote-9)^,^[[10]](#endnote-10)^]. Since informal workers who depend on daily income make up a significant proportion of Nigeria’s population, the lockdown restrictions reduced household incomes especially for the poor. Furthermore, progress with ongoing national and subnational efforts (e.g., social and contributory health insurance schemes) to address existing catastrophic spending have been stalled despite significant progress made in a few states before the pandemic [^[[11]](#endnote-11)^]. Taken together, these challenges further exacerbate high out of pocket and catastrophic expenditure within already vulnerable populations, which has well-documented negative effects on demand for health services.

1. #### Nigeria Center for Disease Control. COVID-19 Nigeria, Confirmed Cases by State. Available at: https://covid19.ncdc.gov.ng

   [↑](#endnote-ref-1)
2. # The Guardian. Nigeria to cut healthcare spending by 40% despite coronavirus cases climbing. 2020 June 10. Available at: https://www.theguardian.com/global-development/2020/jun/10/nigeria-to-cut-healthcare-spending-by-40-despite-coronavirus-cases-climbing?CMP=twt_a-global-development_b-gdndevelopment

   [↑](#endnote-ref-2)
3. IEA (2020), Oil Market Report - April 2020, IEA, Paris https://www.iea.org/reports/oil-market-report-april-2020 [↑](#endnote-ref-3)
4. PWC. Nigeria Economic Alert. Oct 2020. Available at: https://www.pwc.com/ng/en/assets/pdf/economic-alert-october-2020.pdf [↑](#endnote-ref-4)
5. Development Initiatives. Coronavirus and aid data: What the latest DAC data tells us. 17 APRIL 2020. Available at: https://devinit.org/resources/coronavirus-and-aid-data-what-latest-dac-data-tells-us/#section-1-4 [↑](#endnote-ref-5)
6. WHO. COVID-19 significantly impacts health services for noncommunicable diseases. 1 June 2020. Available at: https://www.who.int/news/item/01-06-2020-covid-19-significantly-impacts-health-services-for-noncommunicable-diseases [↑](#endnote-ref-6)
7. Taxaide. 2019 Poverty and inequality report in Nigeria. Available at: https://taxaide.com.ng/wp-content/uploads/2020/05/2019-POVERTY-AND-INEQUALITY.pdf [↑](#endnote-ref-7)
8. Aregbeshola BS, Khan SM. Out-of-pocket health-care spending and its determinants among households in Nigeria: a national study. J Public Health (Berl.) (2020). https://doi.org/10.1007/s10389-020-01199-x [↑](#endnote-ref-8)
9. # The World Bank. COVID-19 National Longitudinal Phone Survey 2020. November 11, 20. Available at: https://microdata.worldbank.org/index.php/catalog/3712

   [↑](#endnote-ref-9)
10. National Bureau of Statistics, 2018. Unemployment And Under-Employment Report 2017 - 2018. 1st ed. [PDF] Abuja: National Bureau of Statistics, pp.3 - 8. Available at: <http://nigerianstat.gov.ng> [Accessed 12 Dec 2020]. [↑](#endnote-ref-10)
11. Muanya C. Accelerating universal health coverage amidst COVID-19. The Guardian. Oct 1, 2020. Available at: <https://guardian.ng/features/accelerating-universal-health-coverage-amidst-covid-19/> [↑](#endnote-ref-11)
