# Supplementary material for: Achieving global mortality reduction targets and universal health coverage: The impact of COVID-19
Source: PLoS Med. 2021 Jun 24;18(6):e1003675. doi: 10.1371/journal.pmed.1003675 (PMC8270396; doi:10.1371/journal.pmed.1003675)
Supplement: S5 Text — COVID-19, Coronavirus Disease 2019; NCD, noncommunicable disease; PHC, primary healthcare. (DOCX) [file pmed.1003675.s007.docx]

### **S5 Text. NCD control in PHC in the COVID era**

Prior to the COVID-19 pandemic, the world was already off track to meet the third Sustainable Development Goal target for NCDs, which specifies a one-third reduction in NCD mortality between 2015 and 2030 [^[[1]](#endnote-1)^]. While many countries observed steady NCD mortality declines in the 1990s and 2000s, there is evidence that progress has slowed since 2010, and for a number of countries and specific NCD causes, age-specific death rates are increasing, raising the prospect of increased global inequality in the future. Reasons for decelerations in progress on NCDs include worsening risk factor trends (especially for emerging risks like obesity) as well as insufficient investment in PHC-based prevention and early treatment of common NCDs.

In many LICs and lower-MICs, NCD programs and divisions in ministries of health have not been sufficiently prioritized or resourced, and NCD capacity-building has not been a priority for DAH. Thus, the disruptions caused by the pandemic will probably derail the NCD agenda even more than the grand convergence agenda. WHO surveys conducted earlier in the pandemic found that NCD services were among those most disrupted by COVID-19, reflecting an implicit rationing of resources towards more urgent needs. Some disruptions, such as suspension of community screening programs and cancellation of elective care (including surgical care) have clearly been necessary to help cope with the pandemic. Others, such as drop-offs in PHC-based delivery of primary and secondary prevention of CVD, or basic treatment of acute cardiac and respiratory diseases, have led to an uptick in excess mortality [^[[2]](#endnote-2)^]. The recommendations of Blanchet and colleagues for essential non-COVID health services underscore the importance of protecting essential NCD interventions within UHC benefits packages. Contrary to popular belief, these interventions are often just as cost-effective as interventions for grand convergence conditions [^[[3]](#endnote-3)^,^[[4]](#endnote-4)^].

Still, the disruptions COVID-19 has created for NCD care provide a unique opportunity to reinvent PHC in LICs and LMICs. In addition to telemedicine (Box 6), which holds promise for expanding access to NCD diagnostic and specialist physician services, other innovations should be explored as part of a comprehensive plan to “move the needle” on NCDs. For instance, drone-based delivery, e-procurement, blockchain and other new technologies, combined with policy reforms, could substantially increase access to high-quality NCD medications, which are often the least available of all the medicines deemed essential for PHC settings [^[[5]](#endnote-5)^]. Mobile health platforms could be leveraged to develop triage and referral tools to close gaps in time-sensitive care, such as drug therapies for acute coronary syndromes [^[[6]](#endnote-6)^]. Above all, the guiding principle at the cross-country level should be collaboration and experimentation with alternative care delivery models that are leaner (in terms of human and financial resources) and easier to scale rapidly while maintaining high quality and patient-centeredness.

While the grand convergence is focused on infections and on maternal and child health conditions, the CIH publications have consistently called for greater action on NCDs. Given the unprecedented pace of demographic and epidemiological change in LICs and LMICs, governments delay action on NCDs at their own peril. By combining intersectoral policies (such as tobacco and alcohol taxes and air pollution controls) with PHC-based preventive care and early treatment, ministries of health can substantially reduce the scale of the NCD epidemic in the future [^[[7]](#endnote-7)^] and thus mitigate much of the fiscal pressure that health and social care systems will face in the absence of urgent action.

1. Bennett JE, Stevens GA, Mathers CD, Bonita R, Rehm J, Kruk ME, et al. NCD Countdown 2030: worldwide trends in non-communicable disease mortality and progress towards Sustainable Development Goal target 3.4. Lancet, Volume 392, Issue 10152, 1072 - 1088 [↑](#endnote-ref-1)
2. Brant LCC, Nascimento BR, Teixeira RA, Lopes MACQ, Malta DC, Oliveira GMM, et al. Excess of cardiovascular deaths during the COVID-19 pandemic in Brazilian capital cities. BMJ Heart. 2020; 106:1898-1905. [↑](#endnote-ref-2)
3. Blanchet K, Alwan A, Antoine C, Cros MJ, Feroz F, Guracha TA, et al. Protecting essential health services in low-income and middle-income countries and humanitarian settings while responding to the COVID-19 pandemic. BMJ Glob Health 2020;5:e003675. Available at: <https://gh.bmj.com/content/5/10/e003675> [↑](#endnote-ref-3)
4. Horton S, Gelband H, Jamison D, Levin C, Nugent R, Watkins D. Ranking 93 health interventions for low- and middle-income countries by cost-effectiveness. PLoS ONE 12(8): e0182951. https://doi.org/10.1371/journal.pone.0182951 [↑](#endnote-ref-4)
5. Center for Global Development. 2019. Tackling the Triple Transition in Global Health Procurement: Promoting Access to Essential Health Products through Aid Eligibility Changes, Epidemiological Transformation, and the Progressive Realization of Universal Health Coverage. Available at: https://www.cgdev.org/sites/default/files/better-health-procurement-tackling-triple-transition-small.pdf [↑](#endnote-ref-5)
6. Watkins D. Cardiovascular health and COVID-19: time to reinvent our systems and rethink our research priorities. Heart. 2020;0:1–3. doi:10.1136/heartjnl-2020-318323 [↑](#endnote-ref-6)
7. Watkins D, Hale J, Hutchinson B, Kataria I, Kontis V and Nugent R. Investing in non-communicable disease risk factor control among adolescents worldwide: a modelling study. BMJ Glob Health 2019;4:e001335. doi:10.1136/ bmjgh-2018-001335 [↑](#endnote-ref-7)
