# Supplementary material for: Achieving global mortality reduction targets and universal health coverage: The impact of COVID-19
Source: PLoS Med. 2021 Jun 24;18(6):e1003675. doi: 10.1371/journal.pmed.1003675 (PMC8270396; doi:10.1371/journal.pmed.1003675)
Supplement: S6 Text — NCD, noncommunicable disease. (DOCX) [file pmed.1003675.s008.docx]

### **S6 Text. How telemedicine could transform NCD care: the example of hearing loss care**

In December 2019, COVID-19 spread rapidly in Wuhan, China. The city was the first to undergo a lockdown, during which 13,000 deaf residents felt they were left behind because they lost access to timely information, goods, and services [^[[1]](#endnote-1)^]. Now, COVID-19 has produced a similar impact on 500 million people with hearing loss worldwide. First, most people with hearing loss are seniors, who are not only the most vulnerable to the virus, but also need hearing services the most in order to stay connected during the pandemic. However, they are unwilling or unable to get the services they need because of the fear of going out or the lockdown of most hearing care centers. Second, those in low- and middle-income countries who rely on community-based services cannot get adequate and prompt hearing services due to overstretched health systems. Last but not least, masks and personal protection equipment make it even harder to hear sounds, and they also block lipreading cues, thereby further aggravating the communication difficulty experienced by people with hearing loss.

Technology, particularly advances in telemedicine, can provide solutions to these challenges facing people with hearing loss during the pandemic and beyond. There is accumulating evidence that, compared with personal and hospital-centered models, tele-care and home-based technologies provide an equivalent level of quality for hearing care and services. These services include basic audiological diagnoses ranging from audiograms to otoscopies as well as advanced treatments such as remotely-fitted hearing aids and tinnitus management [^[[2]](#endnote-2)^,^[[3]](#endnote-3)^,^[[4]](#endnote-4)^,^[[5]](#endnote-5)^]. Not only does tele-care save time and money, but more importantly it addresses the professional shortage issue that has stretched the current health systems, especially in rural areas and low- and mid-income countries [^[[6]](#endnote-6)^]. Rapid development and deployment of allied technologies, such as those capable of compensating for sound attenuation or automatic captioning that can replace lost lipreading cues, can further facilitate communication and usage of tele-medicine for people with hearing loss [^[[7]](#endnote-7)^].

If technology is not a problem, then what are the barriers to prevent the cost-effective and universal implementation of tele-medicine in hearing care and services? One barrier is the digital literacy, or lack thereof, in those who need and can benefit from tele-medicine the most. While it is easy for a young student to download and set up an application, it can be extremely challenging for a senior person who lives in isolation. Moreover, limited access to broad bandwidth or the ability to pay for such bandwidth and other accessories may prevent many in low- and middle-income countries from taking advantage of tele-medicine. Another barrier is reluctance or even resistance from providers and payers. The existing providers may worry about tele-medicine taking away their business while payers are unsure or unwilling to reimburse for the tele-care and services.

The pandemic provides a once-in-a-lifetime opportunity to overcome these barriers and transform global health. COVID-19 forces the health systems, as well as regulatory and insurance agencies, to invest in improving digital literacy and tele-medicine infrastructure. Wide adoption of tele-medicine will lower the cost, reduce existing service disparities between poor and rich areas, and alleviate the tension stemming from overstretched health systems to provide low-cost, high-quality universal health coverage.

1. Clark J, Donai J, Kraus N, Smith K, Sydlowski S, Zeng FG. Audiological Needs, Solutions in COVID-19. The Hearing Journal. 2020;73(6):6-8. [↑](#endnote-ref-1)
2. Sandstrom J, Swanepoel D, Laurent C, Umefjord G, Lundberg T. Accuracy and Reliability of Smartphone Self-Test Audiometry in Community Clinics in Low Income Settings: A Comparative Study. Ann Otol Rhinol Laryngol. 2020;129(6):578-84. Epub 2020/01/23. doi: 10.1177/0003489420902162. PubMed PMID: 31965808. [↑](#endnote-ref-2)
3. Chan J, Raju S, Nandakumar R, Bly R, Gollakota S. Detecting middle ear fluid using smartphones. Science Translational Medicine. 2019;11(492): eaav1102. doi: ARTN eaav1102

   10.1126/scitranslmed.aav1102. PubMed PMID: WOS:000467944100002. [↑](#endnote-ref-3)
4. Convery E, Keidser G, McLelland M, Groth J. A Smartphone App to Facilitate Remote Patient-Provider Communication in Hearing Health Care: Usability and Effect on Hearing Aid Outcomes. Telemed J E Health. 2020;26(6):798-804. Epub 2019/08/23. doi: 10.1089/tmj.2019.0109. PubMed PMID: 31433259; PubMed Central PMCID: PMCPMC7301323. [↑](#endnote-ref-4)
5. Aazh H, Swanepoel W, Moore BCJ. Telehealth tinnitus therapy during the COVID-19 outbreak in the UK: uptake and related factors. Int J Audiol. 2020:1-6. Epub 2020/10/02. doi: 10.1080/14992027.2020.1822553. PubMed PMID: 33000663 [↑](#endnote-ref-5)
6. Bhutta MF. Models of service delivery for ear and hearing care in remote or resource-constrained environments. J Laryngol Otol. 2018:1-10. Epub 2018/12/19. doi: 10.1017/S0022215118002116. PubMed PMID: 30558687. [↑](#endnote-ref-6)
7. McKee M, Moran C, Zazove P. Overcoming Additional Barriers to Care for Deaf and Hard of Hearing Patients During COVID-19. JAMA Otolaryngol Head Neck Surg. 2020;146(9):781-2. Epub 2020/07/22. doi: 10.1001/jamaoto.2020.1705. PubMed PMID: 32692807. [↑](#endnote-ref-7)
